# Quantifying Antimicrobial Use in Dutch Companion Animals

**DOI:** 10.3389/fvets.2019.00158

**Published:** 2019-05-28

**Authors:** Nonke E. M. Hopman, Marloes A. M. van Dijk, Els M. Broens, Jaap A. Wagenaar, Dick J. J. Heederik, Ingeborg M. van Geijlswijk

**Affiliations:** ^1^Department of Infectious Diseases and Immunology, Faculty of Veterinary Medicine, Utrecht University, Utrecht, Netherlands; ^2^The Netherlands Veterinary Medicines Institute (SDa), Utrecht, Netherlands; ^3^Wageningen Bioveterinary Research, Lelystad, Netherlands; ^4^Division Environmental Epidemiology, Institute for Risk Assessment Sciences, Utrecht University, Utrecht, Netherlands; ^5^Pharmacy Department, Faculty of Veterinary Medicine, Utrecht University, Utrecht, Netherlands

**Keywords:** antimicrobial, antibiotic, companion animals, veterinary medicine, defined daily dose, DDDA, prescribing

## Abstract

Antimicrobial resistance (AMR) is an increasing threat, both in human and in veterinary medicine. To reduce the selection and spread of AMR, antimicrobial use (AMU) should be optimized, also in companion animals. To be able to optimize AMU, a feasible method to quantify AMU and information on current AMU are needed. Therefore, a method to quantify AMU was developed, using the number of Defined Daily Doses Animal (DDDA). This method was used to explore applied antimicrobial classes and to identify differences in prescribing patterns in time and between veterinary clinics. Antimicrobial procurement data of the years 2012–2014 were collected retrospectively from 100 Dutch veterinary clinics providing care for companion animals. The mean number of DDDAs per clinic per year decreased significantly from 2012 to 2014. A shift in used classes of antimicrobials (AMs) was seen as well, with a significant decrease in use of third choice AMs (i.e., fluoroquinolones and third generation cephalosporins). Large differences in total AMU were seen between clinics ranging from 64-fold in 2012 to 20-fold in 2014. Despite the relative low and decreasing AMU in Dutch companion animal clinics during the study, the substantial differences in antimicrobial prescribing practices between clinics suggest that there is still room for quantitative and qualitative optimization of AMU.

## Introduction

Antimicrobial resistance (AMR) is an increasing threat, both in human and in veterinary medicine. Many antimicrobials (AMs) used in veterinary medicine are used in human medicine as well. Due to the close contact between people and their companion animals, the importance of companion animals as potential reservoirs of (multi)-resistant pathogens for humans has received increasing attention (1–5). Besides the potential public health threat, AMR also has a direct impact on animal health and welfare, because of treatment failure. To prevent selection and spread of resistant bacteria and to keep AMs valuable for the future, antimicrobial use (AMU) should be optimized.

From 2008 onwards, AMU in Dutch food producing animals received increasing attention, actions were taken at different levels and AMU was reduced considerably (6–9). Most actions addressed food producing animals, but classification of AMs in 1st, 2nd, and 3rd choice AMs [Werkgroep Veterinair Antibiotica Beleid (Working Party for Policy on Veterinary Antimicrobials)]^1^ and legislation on mandatory susceptibility testing for the use of 3rd choice AMs (10) also hold for companion animals.

Risk management of AMR needs to be based on valid and most updated information. Therefore, it is crucial to monitor the amount and types of AMs used in animals. Amounts and types of AMs used in animals have been investigated in several countries, particularly in food producing animals (11–16). Only a few studies describe AMU and prescribing patterns in companion animals (17–21). The majority of studies regarding AMU in companion animals uses total sales or prescription data expressed in kilograms of AMs (18), the mass of active AM substances (by AM class or subclass) in relation to a specified population to express AMU or the number of prescriptions (15, 20–22). These different measurement units make it hard to compare data between these studies. The European Medicines Agency, European Surveillance of Veterinary Antimicrobial Consumption group (EMA ESVAC) has introduced the veterinary Defined Daily Doses for Animals (DDD_VET_) to objectify the numerator (23, 24). DDD_VET_ is defined as a “technical unit of measurement similar to the Defined Daily Dose (DDD), usually based on recommendations from the Summary of Product Characteristics (SPC) and in some cases based on scientific literature, intended for the purpose of drug consumption studies. DDD_VET_ is assigned per kilogram animal per species per day” (23, 24). According to ESVAC, objective AMU data collection should also be organized for companion animals, rabbit production and aquaculture (25).

The aim of present study was to quantify systemic AMU in Dutch companion animal clinics (2012–2014) using Defined Daily Doses Animal (DDDA) established according to the Dutch authorization of the veterinary medicinal products, to explore applied antimicrobial classes and to identify differences in prescribing patterns in time and between veterinary clinics.

## Materials and Methods

### Study Design and Data Collection

A retrospective survey was performed. The Royal Dutch Veterinary Association (KNMvD) provided contact details of all 1,149 veterinary clinics in the Netherlands which treated companion animals. All these clinics were invited by mail to participate, followed by a reminder after 2 weeks by e-mail. Requested data were clinic population data and antimicrobial veterinary medicinal product (AVMP) procurement data for the subsequent years 2012, 2013, and 2014. Mixed-animal clinics with combined, unspecified procurement data for companion animals and non-companion animals were excluded from the study, because products with a multi-species (companion and food producing animal) registration could not be allocated to specific animal species.

### Calculation of DDDAs

In the Netherlands, AMs for veterinary use are on prescription only and sold to companion animal owners (or farmers) by veterinarians exclusively. Therefore, antimicrobial procurement data are supposed to reflect the total amount of AMs used in animals. These procurement data were used to calculate the number of DDDAs per clinic per year (DDDA_CLINIC_). For each year and clinic, the number of ordered packages of AVMPs for systemic use was provided, identified by their unique European Article Number (EAN)-code. To calculate the number of DDDAs per clinic, two variables are needed (13). First, the total animal mass in kilogram that can be treated for 1 day with the amount of AMs prescribed; for every individual AVMP this can be derived from the “DG-standaard” by EAN-code. The DG-standaard is an online Dutch database containing all packages of AVMPs once authorized in the Netherlands, managed by the Netherlands Veterinary Medicines Institute. For every single AVMP package, per species the total animal mass in kilogram that can be treated is defined, preferably based on authorized doses, for cascade use based on comparable AVMPs or literature [SDa (the Netherlands Veterinary Medicines Institute)]^2^. This database was initially developed and applied for the monitoring of antimicrobial consumption in the major food producing animal sectors, enabling e.g., benchmarking of farms within sectors. Second, the total weight (in kg) of the clinic animal population at risk to be treated with the AVMP. The latter was estimated based on the clinic animal population represented by the number of dogs, cats and rabbits attending the clinic at least once in a specified 3-year period. The total weight was calculated by multiplying the number of dogs, cats and rabbits with previously established average body weights for dogs (19.1 kg) and cats (4.1 kg) (26), for rabbits the average weight was based upon expert opinion (2.5 kg). For every AVMP, the denominator was determined separately depending on the animal species the AVMP was authorized for. By dividing the two variables for all individual AVMPs and consequently adding up the outcomes, the total number of DDDAs is obtained. This sum of all AVMPs is suitable for comparison between clinics and between consecutive years (DDDA_CLINIC_).

This calculation results in the indicator DDDA_CLINIC_/year that represents the theoretical number of days per year an average animal (dog, cat or rabbit) was treated with AVMPs in the clinic concerned. For example, a DDDA_CLINIC_ in 2014 of 2 implies that the average dog, cat and rabbit in care of this veterinary clinic has received 2 days of AM-treatment in 2014.

### Classification of AMU

Classification of AMU in present study (Table 1) is according to the Dutch policy on veterinary AMU [Werkgroep Veterinair Antibiotica Beleid (Working Party for Policy on Veterinary Antimicrobials)]^1^.

**Table 1:** Classification of veterinary antimicrobials (AMs) in 1st, 2nd, and 3rd choice AMs, according to Dutch policy on veterinary AMU.

**Classification**	**Reasoning**	**Main classes of AMs**
1st choice	Empirical therapy; do not select for (to current knowledge), nor are specifically meant for treatment of ESBL-producing micro-organisms	Tetracyclines, nitroimidazoles, narrow-spectrum penicillins, trimethoprim, sulfonamides, and phenicols
2nd choice	All AMs not classified as 1st or 3rd choice AMs; Use of these AMs might select for ESBL-producing bacteria or is specifically indicated in case of an ESBL-infection	Aminopenicillins (with/without beta-lactamase inhibitors), 1st generation cephalosporins, aminoglycosides and colistin
3rd choice	Highest priority critically important AMs for human medicine according to WHO; By Dutch law restricted to use only in individual animals and after culture and susceptibility testing	Fluoroquinolones, 3rd and 4th generation cephalosporins

### Statistical Analysis

DDDA_CLINIC_ data were used to determine the proportion of 1st, 2nd, and 3rd choice AMs, to identify trends in AMU during the study period and to identify differences between clinics. Mixed models were used to explore the variation in AMU over time, both within and between clinics. Models for AMU (total, 1st, 2nd, and 3rd choice) were fitted using PROC GLIMMIX (SAS 9.4, SAS Institute, Inc., Cary, NC, USA) assuming a log-normal distribution and allowed for changes in residual variance over time. Within clinic correlations were modeled using an autoregressive [ARH(1)] model and a random intercept. The year of prescription was included as a categorical covariate and statistical significance was tested for using likelihood ratio-testing, comparing model fit to that of a model that not included this covariate (both fitted using maximum likelihood). *P* < 0.05 were considered statistically significant.

## Results

### Inclusion of Clinics

In total, 155 veterinary clinics responded and were willing to provide specified antimicrobial procurement data (13.5% of the total number of invited clinics). Because of missing data or Practice Management System (PMS) incapability to properly report the animal population data, 44 clinics were excluded. Procurement data from 111 veterinary clinics (period 2012–2014) were included and analyzed. Data from 11/111 veterinary clinics turned out to be inconsistent or unrealistic, i.e., reporting an unexpectedly high or low number of dogs or cats (about 10-times higher or lower than the average clinic) or AVMPs for food producing animals appeared to be incorrectly ascribed to companion animals. Therefore, results are based on data of 100 participating clinics.

### Antimicrobial Use: Changes Over Time and Differences Between Clinics

**Figure 1:**
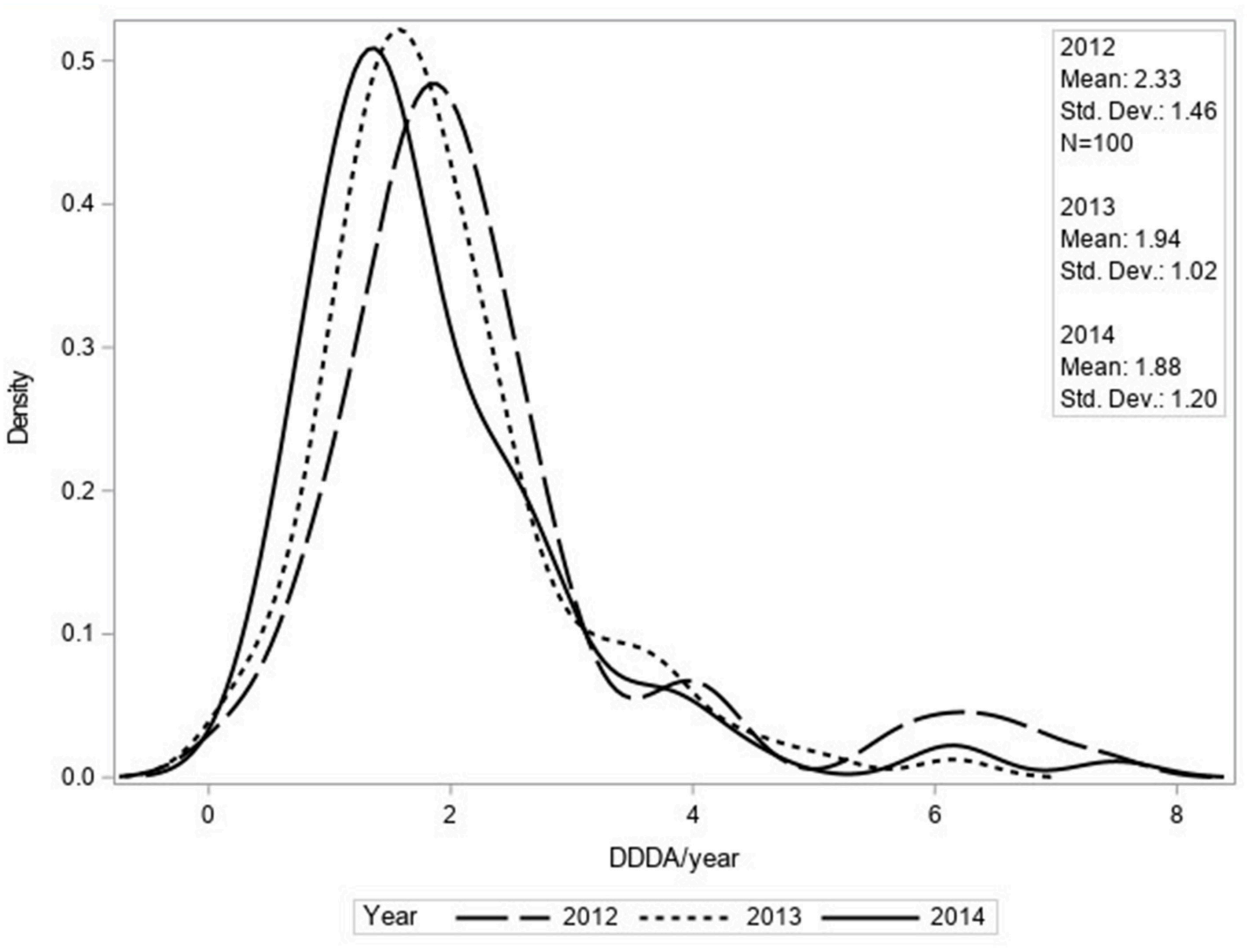
Density function of DDDA_CLINIC_/year for total AMU based upon procurement data of 100 clinics for 2012, 2013, and 2014.

**Figure 2:**
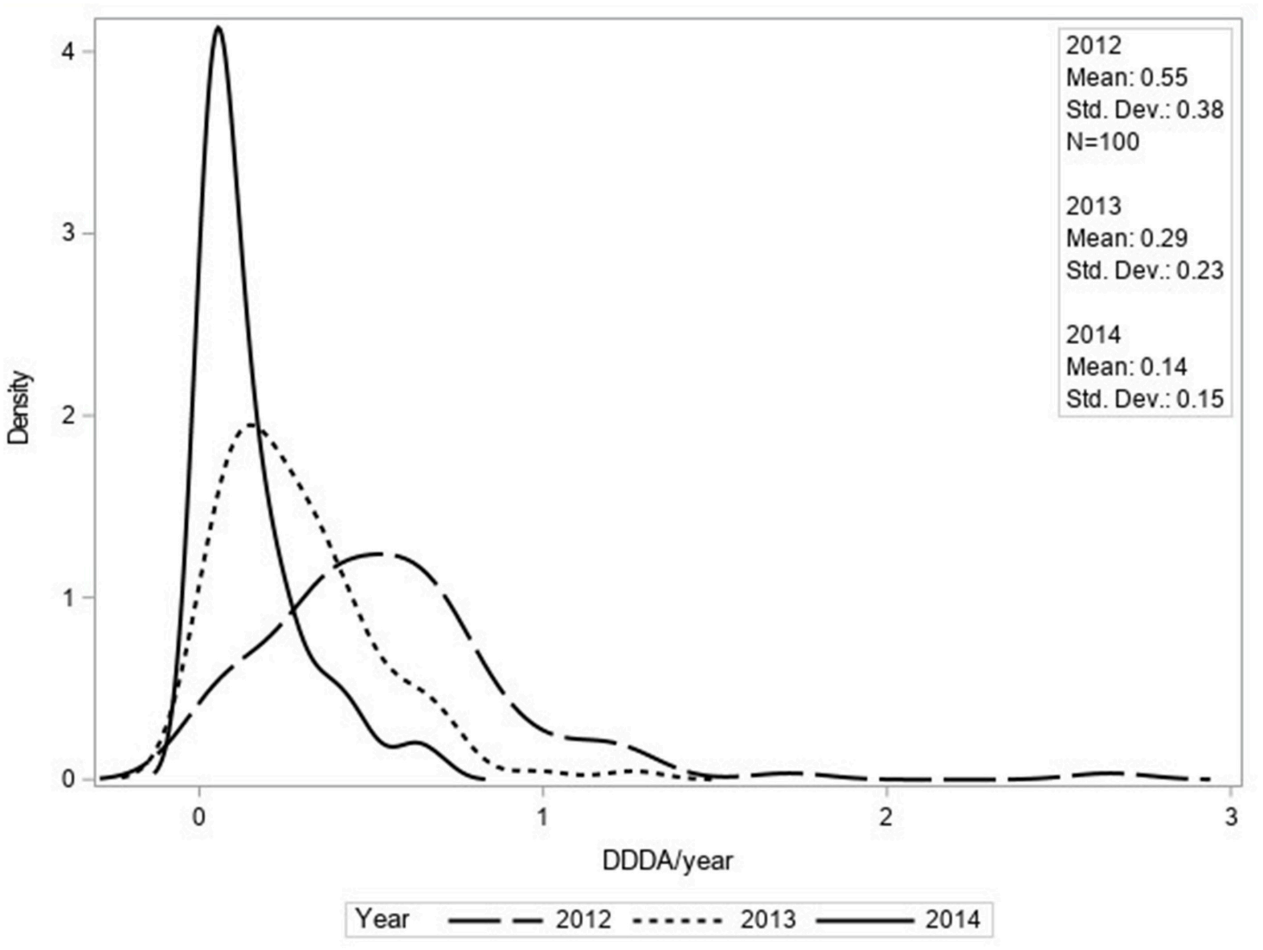
Density function of DDDA_CLINIC_/year for third choice AMU based upon procurement data of 100 clinics for 2012, 2013, and 2014.

The mean number of DDDAs per clinic per year (DDDA_CLINIC_) decreased from 2.33 (±1.46) in 2012 to 1.88 (±1.20) in 2014 (Figure 1). Use of 2nd choice AMs also decreased during the study period [0.97 (±0.77) in 2012 to 0.81 (± 0.63) in 2014] as was the case for 3rd choice AMs [0.55 (±0.38) in 2012 to 0.14 (±0.15) in 2014] (Figure 2). First choice AMU increased from 0.81 (±0.93) in 2012 to 0.93 (±0.71) in 2014. Mixed model analyses of AMU (log-transformed data) indicated that all differences between 2012 and 2014 were statistically significant.

In 2012 and 2013, 2nd choice AMs were the most frequently used compounds (42 and 46% of total AMU), whereas in 2014, 1st choice AMs were most frequently used (50% of total AMU). With regard to the groups of AMs used, aminopenicillins (with or without clavulanic acid) defined as 2nd choice AMs, represented the largest group in all three consecutive years (2012; 31%, 2013; 36% and 2014; 36% of total AMU). In 2012, the second largest group of AMs consisted of 3rd generation cephalosporins (i.e., cefovecin) (14% of total AMU), in 2013 and 2014 the second largest group consisted of trimethoprim/sulfonamides (11 and 13% of total AMU, respectively) which are 1st choice AMs. The use of fluoroquinolones and 3rd generation cephalosporins (both 3rd choice AMs) decreased from a mean DDDA_CLINIC_/year number of 0.22 and 0.33 (2012) to 0.08 and 0.07 (2014), respectively.

The majority of systemically used AMs were orally administered (2012 66%; 2013 73%; 2014 77%, respectively). However, major part of 3rd choice AMs were applied parenterally (2012 67%; 2013 63%; 2014 55%, respectively), although this distribution is shifting toward more oral use as well.

**Figure 3:**
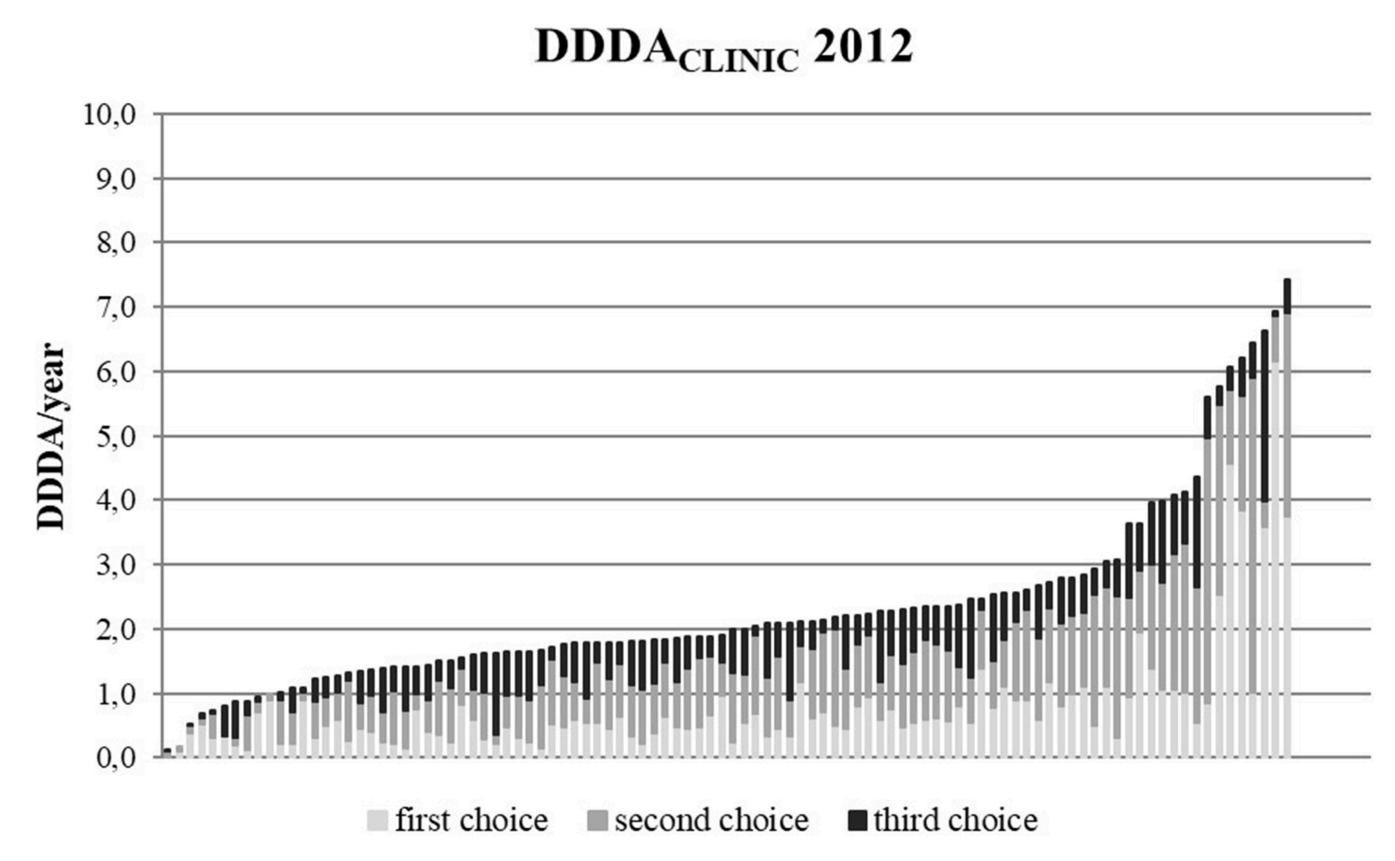
DDDA_CLINIC_ figures for all 100 clinics in 2012, specified for first, second, and third choice antimicrobials, showing the differences in AMU between clinics (based upon procurement data of these 100 clinics).

**Figure 4:**
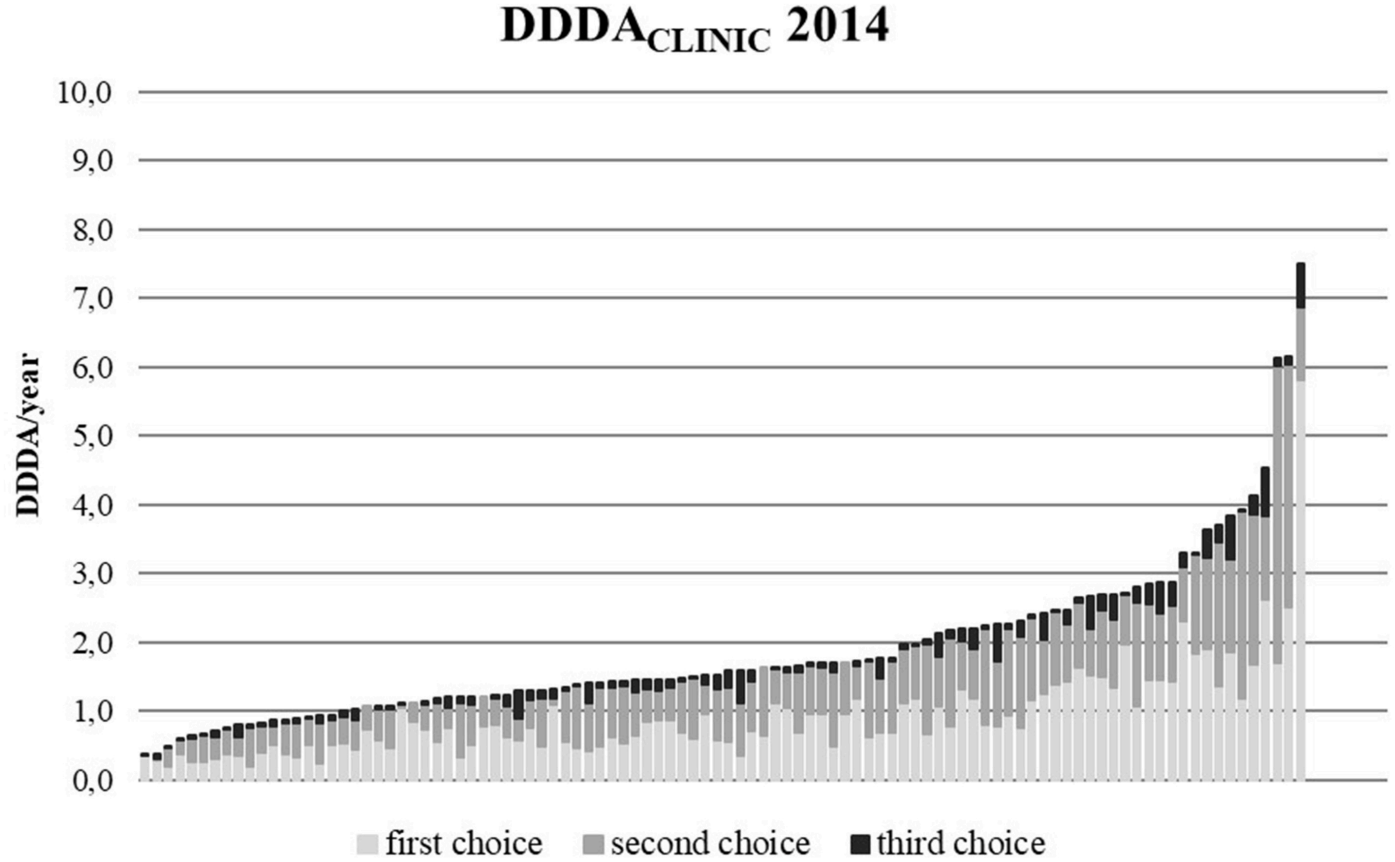
DDDA_CLINIC_ figures for all 100 clinics in 2014, specified for first, second, and third choice antimicrobials, showing the differences in AMU between clinics (based upon procurement data of these 100 clinics).

The DDDA_CLINIC_ numbers varied from year to year and per clinic (Figures 3, 4). From 2012 to 2014, overall DDDA_CLINIC_ numbers from individual clinics ranged from 0.11 (minimum DDDA_CLINIC_, 2013) to 7.5 (maximum DDDA_CLINIC_, 2014). In 2012, the between clinic difference in total AMU was almost 64-fold (Figure 3). In 2014, the between clinic difference was smallest and amounted a 20-fold difference between the minimum and maximum DDDA_CLINIC_ (0.37–7.50) (Figure 4). An interesting detail in this observation is that a higher minimum DDDA_CLINIC_ caused the drop in the between clinic difference, not a lower maximum DDDA_CLINIC_. Spearman correlations between repeated measures of total AMU for different pairs of years ranged between 0.7 and 0.8. Regarding the use of 3rd choice AMs, the between clinic difference was larger. Five clinics reported no 3rd choice AMU in 2014. The lowest use that was reported accounted for a DDDA_CLINIC_ of 0.001 while the maximum use was 0.70 in the same year, accounting for a 500-fold difference in 3rd choice AMU between clinics in 2014.

Statistical modeling established the observed differences in AMU between clinics by the mixed model analyses of AMU (log-transformed data) with a heterogeneous AR(1) model, a random “clinic” effect and year of prescription as a covariate. For total AMU the residual variances decreased by 26% from 2012 to 2014. However, for 3rd choice AMU the residual variances increased by 102%, indicating that differences between clinics for the use of these AMs became more prominent over time. The estimated correlation between residuals of repeated measures of total AMU for a single clinic for different pairs of years ranged from 0.62 to 0.77 (using log-transformed data), indicating that clear systematic differences exist between practices in AMU.

## Discussion

The present study used the number of DDDAs per clinic to express AMU in companion animals. By applying DDDAs dosing differences between AMs due to e.g., the relative potency and differences in pharmacokinetics, are taken into account, as well as dosing differences between species. This measure enables objectified comparison over time and between clinics, even internationally. This measure is adopted in monitoring AMU in food producing animals and endorsed by EMA ESVAC (23). Despite the advantages of a more harmonized way of presenting AMU, there are some disadvantages as well. Disadvantages of using DDDAs are linked to the way DDDAs are calculated. Two variables are needed for this calculation (13) (1) a numerator expressing the total treated animal weight and (2) a denominator expressing the total weight of the clinic animal population. Both variables might be biased. For the numerator this might be the case when an AVMP is authorized for use in more than one animal species. The majority of AVMPs in this study is authorized for more than one of the companion animal species concerned and due to lacking prescription information, it could not be specified whether these AVMPs were prescribed to dogs, cats, and/or rabbits. When it is unknown whether the product has been administered to dogs, cats, or rabbits, the resulting DDDA_CLINIC_ cannot be stratified to specific animal species. At the same time, to be able to determine the total treated animal weight in case of an AVMP that is authorized for more than one companion animal species, the numerator was calculated using the average number of kilograms treated of the species the AVMP was registered for. As an example, if an AVMP was authorized for both dogs, cats and rabbits, the average number of treated kilograms of dogs, cats, and rabbits was calculated as the numerator. In food producing animals, prescription data are collected on farm level making it easier to allocate the AVMPs to specified animal species. Only prescription data (identifying the animal the AVMP was prescribed for) can mitigate this problem of AVMPs authorized for more than one companion animal species. For the denominator, bias might be caused by the total weight of the different animal species and the clinic animal population represented by the number of dogs, cats, and rabbits attending the clinic at least once in a specified 3-year period. In our study, the total clinic animal population of all 100 participating clinics consisted of 228,000 dogs, 228,000 cats, and 25,000 rabbits. These 100 clinics represented 8.7% of 1,149 veterinary clinics treating companion animals in the Netherlands. When these numbers are extrapolated and compared to official estimates on the number of dogs, cats and rabbits in the Netherlands (27), the total number of dogs in the Netherlands is overestimated (correction factor 0.57), the number of cats seems to be estimated correctly (correction factor 0.99) and the number of rabbits is underestimated (correction factor 4.13). The discrepancies between the number of dogs and rabbits registered in veterinary clinics vs. official estimates in the Netherlands (based upon a survey among 7,500 Dutch households) might be explained by the fact that rabbit owners consult a veterinarian less often and dog owners might visit more different clinics (e.g., for a second opinion). The relatively high number of dogs compared to rabbits, might also be explained by the fact that rabbit owners mainly visit a veterinarian in case a rabbit is ill, while dog and cat owners might also seek preventive veterinary medicine (e.g., yearly check-ups, vaccinations etc.).

Additional calculations taking above mentioned correction factors into account, result in a mean overall AMU of 2.8 DDDA/year in 2012 (vs. 2.33 without correction factors), 2.34 DDDA/year in 2013 (vs. 1.94 without correction factors), and 2.27 DDDA/year in 2014 (vs. 1.88 without correction factors). Third choice AMU accounted for a mean DDDA/year of 0.69 in 2012 (vs. 0.55), 0.36 in 2013 (vs. 0.29), and 0.19 in 2014 (vs. 0.14), respectively. Although absolute DDDA_CLINIC_ numbers are higher using the correction factors, observed trends and patterns in AMU and differences between clinics remain the same. Regarding the applied denominator per clinic, the absolute DDDA_CLINIC_ values should be interpreted with caution. DDDA is a powerful and objective measure. For comparisons over time and between studies, the denominator should be well-defined.

This study shows a significant decrease of AMU from a mean DDDA_CLINIC_ of 2.33 DDDA/year in 2012 to 1.88 in 2014. This decrease was combined with a clear shift in classes of AMs used. Increased attention for AMU in general and national action plans to establish reduction of AMU in food producing animal sectors, appeared to have affected AMU in Dutch companion animals as well. Not only in the Netherlands, but also in other countries a recent decrease in AM prescriptions in companion animals was reported (20, 21). However, in present study considerable differences in AMU between clinics were seen, suggesting possibilities for optimization of AMU. Given the observation that repeated measures of total AMU from one specific clinic were clearly correlated and substantial between-clinic differences were observed, it would be worth focusing on those clinics with less favorable figures first, although differences between clinics reduced with decreasing use as well. Because 3rd choice AMU was already relatively low, yearly use tended to fluctuate more. Therefore, repeated measures of 3rd choice AMU from one specific clinic appeared less correlated.

Despite a significant reduction in total AMU and especially in 3rd choice AMs [CIAs of highest priority for human medicine according to WHO (28)], the use of these AMs still accounted for 7.7% of total AMU in 2014. However, hard to compare due to using different measurements of AMU, other countries report similar or slightly higher use of highest priority CIAs: in the UK, CIAs of highest priority accounted for just over 6% of AMs used in dogs and 34% in cats (calculated as number of events) (18) and in Australia 8% of the AM courses prescribed belonged to CIAs of highest priority, in which cats were 4.8-times more likely than dogs to receive 3rd generation cephalosporins (21).

Second choice AMs (mainly aminopenicillins and 1st generation cephalosporins) represented the AMs most frequently used in studied Dutch companion animal clinics in 2012 and 2013. Aminopenicillins are categorized as CIAs with a high priority for human medicine (28). These findings are in line with studies in other countries (18, 21).

Total AMU in companion animals is decreasing and relatively low compared to livestock [e.g., in 2014 DDDA_NAT_ for cattle was 2.44, 21.15 for veal calves and 9.52 for pigs, respectively (29)] and AMU in humans [total AMU in the primary care sector of 10.58 DDD/1000 inhabitant days in 2014, corresponding to 3.86 DDD/inhabitant year (30)]. However, regarding the potential selection of ESBL-producing bacteria and regarding the use of 3rd choice AMs, there seems to be room for improvement in the classes and subclasses of AMs used in companion animals. Focus should be on further reduction of 2nd and 3rd choice AMU.

Since January 2013, use of 3rd choice AMs as well as AMs authorized for human use is discouraged by legislation (susceptibility testing is mandatory). Therefore, the amount of AMs authorized for human use used in veterinary medicine is expected to be low. Based on the present study with veterinary wholesalers' procurement data, the exact amount of AMs authorized for human use (e.g., nitrofurantoin, some clindamycin, and trimethoprim/sulfonamide products) could not be calculated, because data from human pharmacies was not included.

Remarkable differences in AMU between clinics were observed. Overall AMU differed 20-fold in 2014, while for 3rd choice AMs this difference was 500-fold. The residual variance for 3rd choice AMU increased, indicating that differences between clinics with regard to 3rd choice AMU became more prominent. In human primary care the difference in number of antimicrobial courses between Dutch practices was only 5-fold (31). Observed differences in present study might partially be attributed to differences in animal population between clinics. E.g., when clinics treat mainly small or very large breeds, the standardized average animal species weights used for DDDA calculations might not be correct and might cause under- or overestimation of the DDDA_CLINIC_. Also differences in first opinion clinics vs. referral (i.e., secondary or tertiary) clinics, or clinics mainly treating emergency patients might account for observed differences between clinics. However and probably more important, AMU will be determined by prescribing policy and habits within companion animal clinics (e.g., the introduction and implementation of current guidelines regarding AMU) and veterinarian related prescribing habits, e.g., personal preferences in used dosages, frequency of dosing and course lengths as was shown in previous qualitative studies on AMU in companion animal clinics (32–34). The observation of clear and systematic differences between clinics in AMU highlights a potential for further optimization of AMU, eventually leading to smaller differences in AMU between clinics. Therefore, it is of interest to explore underlying factors which may explain differences in AMU between clinics in future studies more in-depth.

Only 8.7% of the 1,149 veterinary clinics treating companion animals were enrolled in present study. The representativeness of these clinics for all Dutch companion animal clinics might be questioned. Participating clinics might have had special interest in AMU and therefore display a more responsible attitude in their AMU compared to non-participating clinics. On the other hand, large differences in AMU between clinics could be observed, indicating that not only clinics with a low AMU were participating. Furthermore, participating clinics were distributed over the whole country. Therefore, the authors believe that the patterns of antimicrobial prescribing are likely to reflect those of the greater population and absolute DDDA numbers can be assumed to provide a reliable lower estimate of AMU across the remainder of the Dutch population of companion animal veterinary clinics.

In conclusion, systemic AMU in Dutch companion animal clinics is decreasing, in particular the use of 3rd choice AMs. However, substantial differences in AMU between clinics could be observed, both in (sub) classes as well as in total amount of AMs used, showing room for improvement.

## Data Availability

The raw data supporting the conclusions of this manuscript will be made available by the authors, without undue reservation, to any qualified researcher.

## Contribution to the Field

AMR is recognized in human and in veterinary medicine as an increasing threat. The close contact between man and companion animals justifies the recognition of the importance of companion animals as potential reservoirs of (multi)-resistant pathogens for humans. AMR might have a direct impact on animal health and welfare as well. To prevent selection and spread of resistant bacteria and to keep AMs working and effective in the future, AMU should be optimized. In present study, a method was developed, using the number of DDDA, to quantify AMU. With this method used antimicrobial classes were explored and differences in prescribing patterns in time and between veterinary clinics were described. AMU was relatively low and decreasing in participating companion animal clinics, however substantial differences in prescribing practices between clinics suggest that there is still room for quantitative and qualitative improvement. The applied quantification method enables objectified comparison of AMU over time and between clinics, even internationally. Gathered data and developed quantification method will be used in future studies to explore AMU in Dutch companion animals more in-depth, to inform policy makers on AMU developments and to optimize AMU in companion animals.

## Author Contributions

DH, JW, MvD, and IvG contributed to the concept and design of the study. MvD and IvG collected the data. NH and IvG performed the data analysis. All authors contributed to the writing and revising process of the manuscript.

## Conflict of Interest Statement

The authors declare that the research was conducted in the absence of any commercial or financial relationships that could be construed as a potential conflict of interest.

## Footnotes

^1^WVAB-richtlijn classificatie van veterinaire antimicrobiële middelen. Available: https://www.wvab.nl/

^2^DG-standaard (Dutch database for AVMPs). Available: https://cdn.i-pulse.nl/autoriteitdiergeneesmiddelen/userfiles/Publicaties/sda-standard-operating-procedure-dg-standaard-januari-2015.pdf
